# Strategies to optimise the health equity impact of digital pain self-reporting tools: a series of multi-stakeholder focus groups

**DOI:** 10.1186/s12939-024-02299-w

**Published:** 2024-11-11

**Authors:** Syed Mustafa Ali, Amanda Gambin, Helen Chadwick, William G. Dixon, Allison Crawford, Sabine N. Van der Veer

**Affiliations:** 1 Centre for Health Informatics, Division of Informatics, Imaging and Data Science, Manchester Academic Health Science Centre, The University of Manchester, Manchester, UK; 2 Applied Research Collaboration – Greater Manchester (ARC-GM), National Institute for Health and Care Research (NIHR), Manchester, UK; 3 Centre for Addiction and Mental Health, Toronto, ON Canada; 4 Centre for Epidemiology Versus Arthritis, Division of Musculoskeletal and Dermatological Sciences, Manchester Academic Health Science Centre, The University of Manchester, Manchester, UK; 5 NIHR Manchester Biomedical Research Centre, Manchester University NHS Foundation Trust, Manchester, UK; 6 Northern Care Alliance NHS Foundation Trust, Salford, UK; 7 Department of Psychiatry, University of Toronto, Toronto, ON Canada

**Keywords:** Health equity, Pain assessment, Pain management, Digital health, Digital pain self-reporting tools

## Abstract

**Background:**

There are avoidable differences (i.e., inequities) in the prevalence and distribution of chronic pain across diverse populations, as well as in access to and outcomes of pain management services. Digital pain self-reporting tools have the potential to reduce or exacerbate these inequities. This study aimed to better understand how to optimise the health equity impact of digital pain self-reporting tools on people who are experiencing (or are at risk of) digital pain inequities.

**Methods:**

This was a qualitative study, guided by the Health Equity Impact Assessment tool—digital health supplement (HEIA-DH). We conducted three scoping focus groups with multiple stakeholders to identify the potential impacts of digital pain self-reporting tools and strategies to manage these impacts. Each group focused on one priority group experiencing digital pain inequities, including older adults, ethnic minorities, and people living in socio-economically deprived areas. A fourth consensus focus group was organised to discuss and select impact management strategies. Focus groups were audio-recorded, transcribed verbatim, and analysed using a framework approach. We derived codes, grouped them under four pre-defined categories from the HEIA-DH, and illustrated them with participants’ quotes.

**Results:**

A total of fifteen people living with musculoskeletal pain conditions and thirteen professionals took part. Participants described how digital pain self-reports can have a positive health equity impact by better capturing pain fluctuations and enriching patient-provider communication, which in turn can enhance clinical decisions and self-management practices. Conversely, participants identified that incorrect interpretation of pain reports, lack of knowledge of pain terminologies, and digital (e.g., no access to technology) and social (e.g., gender stereotyping) exclusions may negatively impact on people’s health equity. The participants identified 32 strategies, of which 20 were selected as being likely to mitigate these negative health equity impacts. Example strategies included, e.g., option to customise self-reporting tools in line with users’ personal preferences, or resources to better explain how self-reported pain data will be used to build trust.

**Conclusion:**

Linked to people’s personal and social characteristics, there are equity-based considerations for developing accessible digital pain self-reporting tools, as well as resources and skills to enable the adoption and use of these tools among priority groups. Future research should focus on implementing these equity-based considerations or strategies identified by our study and monitoring their impact on the health equity of people living with chronic pain.

**Supplementary Information:**

The online version contains supplementary material available at 10.1186/s12939-024-02299-w.

## Background

Chronic pain is a global health problem. It affected approximately 1.71 billion people in 2019 [16], with the prevalence and impact continuing to increase. However, certain individuals or groups across countries are further affected because of their personal characteristics and circumstances [14, 44]. For example, in the United Kingdom, older adults, women, gender-diverse individuals, indigenous and other ethnic minority groups, and people living in economically deprived areas are affected more than their younger, male, white and affluent counterparts [28, 36, 56, 58, 75]. In addition to differences in pain prevalence and impact between sub-groups, further disadvantage may result from differences in access to pain management and outcomes [58]. For example, opioid use and opioid-related deaths are higher among people living in deprived areas [2], response to analgesics is poorer among women [11, 22, 44]; access to healthcare services is delayed among ethnic minorities [68], and older adults are less likely to experience good pain management [40]. This suggests that differences in pain prevalence, treatment and outcomes are associated with socio-demographic characteristics, which in turn implies they are unfair and should therefore be considered pain inequities [58].

Pain inequities may partly be due to poor understanding of pain aetiology, its underlying causes, and its impact on people’s lives. Poor quality communication about these pain aspects between patient and providers explains part of this, particularly the lack of summarised pain information available during consultations [38, 49]. In absence of pain information directly collected from patients, providers’ biases may come at play causing delayed diagnosis [68], poor treatment decisions, and suboptimal outcomes [64, 78]. Therefore, better pain assessment and management approaches may help avoid or mitigate these issues for groups experiencing pain inequities, henceforth referred to as priority groups.

Pain self-reporting is central to delivering person-centred, effective pain management services [21, 23]. Therefore, to address pain inequities, it is important that pain self-reporting tools are acceptable and feasible across populations, including priority groups [17, 27, 54]. However, current pain self-reporting tools may put certain groups at a disadvantage because of challenges related to physical and cognitive impairments, language requirements, or pain beliefs [3, 6, 39, 59]. Patients prefer digital self-reporting tools over traditional paper-based questionnaires [49], particularly those with language requirements [25] and several such tools have been developed and tested in the context of pain [5, 4, 53]. However, the digital nature of these tools may inadvertently worsen or cause additional equity gaps: for instance, lower rates of digital literacy, different pain beliefs, and unique accessibility requirements among older adults [3, 6, 43, 49] may negatively impact the use of digital tools among these groups [18].

Although several priority groups have been identified for pain assessment and management, it remains unknown how these priority groups would be impacted by the introduction of digital pain self-reporting tools to facilitate pain management. Therefore, this work aimed to better understand how to optimise the health equity impacts of digital pain self-reporting tools among those who are experiencing or are already at risk of pain inequities. The specific objectives are to:

1. Explore the potential impacts of introducing digital pain self-reporting tools to facilitate pain management on groups at risk of digital pain inequities.
2. Identify and select strategies to optimise these potential equity impacts.

## Methods

This qualitative study involved a series of three online scoping focus groups with multiple stakeholders to identify groups at risk of digital pain inequities, the potential impacts of introducing digital pain self-reporting tools for these groups and strategies to mitigate negative impacts, and a fourth consensus focus group to select the most promising strategies. We reported the methods and results of this study in accordance with the consolidated criteria for reporting qualitative research (COREQ) [72] (Annexure A). The study received an ethical approval from the University of Manchester’s Research Ethics Committee (ref # 2022–14094-23756).

### Digital health equity impact assessment approach

The Health Equity Impact Assessment—Digital Health Supplement (HEIA-DH) is a decision support tool developed to provide more systematic guidance on the inclusion of health equity considerations in the design, development and implementation of digital health technologies and care. Adapted from the Health Equity Impact Assessment Tool (HEIA) developed by the Ministry of Health and Long-Term Care in Ontario, Canada [50], the HEIA-DH aims to engage anyone involved in the development or delivery of digital health technologies and care in adopting a systematic approach to consideration of: (a) scoping what is known about social and digital determinants of health for a given community (in our case, priority groups living with chronic pain), (b) identifying potential unintended equity impacts, (c) strategies for mitigation of unintended negative equity impacts, (d) monitoring these mitigation strategies and, (e) disseminating effective strategies [19].

### Study participants and recruitment

#### Initial selection of priority groups

As part of the first phase of applying the HEIA-DH process, we reviewed the literature and identified the following three priority groups who may experience digital and/or pain inequities in the UK: older adults, ethnic minority groups, and people living in socio-economically deprived areas (Table 1); we organised one scoping focus group for each of them.

**Table 1:** Evidence supporting the initial selection of priority groups experiencing (digital) pain inequities in the UK

Older adults	Ethnic minority groups - (South Asians; black African)	People living in more socio-economically deprived areas
• Higher pain prevalence [61] • Under-reporting of pain due to physical or mental impairment [59] • Differences in pain perception and beliefs [39] • Less likely to use digital health technologies [43]	• Higher pain prevalence [7, 61, 46]) • Poor digital literacy [1] • Less likely to use digital health technologies [43] • Lower completion of daily digital pain reports [3, 6] • Cultural influence on pain self-reporting [3, 6]	• Higher bodily pain levels [65] • More likely to report chronic pain [61] • Poor digital literacy [60]

### Participant eligibility and recruitment

#### Series of multi-stakeholder scoping focus groups

For each of the three scoping focus groups, we invited up to six people living with a musculoskeletal pain condition, complemented by up to four professionals with expertise across relevant fields. The purpose of organising multi-stakeholder focus groups was to capture breadth of views and to co-create practical and relevant insights by complementing patients’ views with professionals’ views for digitally-enabled pain assessment and management.

Adults (18 years or older) living with a musculoskeletal pain condition, representing at least one of the priority groups in Table 1, and able to participate online were eligible to take part in one of the first three focus groups. Through a mix of purposive and convenience sampling, we approached people by sharing a study flyer (Annexure B) with those who had participated in a previous, related study [3, 6] and via online groups of people with an interest in taking part in research studies. We purposively invited people from a previous, related study because participants with personal experience of using the technology may find it easier to articulate and communicate their thoughts and may provide more in-depth perspectives by reflecting upon their experience [55].

We approached potential professional participants through our own professional networks. They were eligible if they provided health services to people living with musculoskeletal pain conditions or if they had other relevant domain expertise (e.g., in healthy ageing, digital inclusion, technology development, chronic pain, health equity).

All participants completed an online consent form before the start of a focus group. As an example of a digital pain self-reporting tool, we encouraged participants to download the Manchester Digital Pain Manikin app on their smartphones [3, 6, 73] and/or watch a video showing how to self-report their pain using the app (see Table 2 for details). Although we could not mitigate all barriers to participating in a virtual focus group, participants were given the opportunity to join a 1-on-1 technical support session prior to the focus group for setting up the virtual meeting platform on their devices. Patient participants received a gift voucher to acknowledge their contribution.

**Table 2:** Manchester Digital Pain Manikin – example of a digital pain self-reporting tool (copyright University of Manchester)

The Manchester Digital Pain Manikin is a digital pain self-reporting tool on which people can indicate the location and intensity of their pain by drawing directly on the front and back view of a two-dimensional, gender-neutral human-shaped figure. The app is an improved and tested version of a previous prototype [2, 6, 73]. A daily pain manikin report includes a single overall pain question, a two-sided two-dimensional pain drawing, and a free text pain diary (Fig. 1a-d). The app enables zooming in on a specific part of the body by selecting an area from a pre-specified list.			
			
1a. Numeric rating scale for overall pain intensity	1b. Front view of the body manikin with pain drawing	1c. Back view of the body manikin with pain drawing	1d. Pain diary

To help manage potential imbalances in power between different stakeholder groups (e.g., between professionals and people with lived experience), we shared a set of ground rules with all participants on the day of focus group. The ground rules emphasised maintaining respect between people living with chronic pain and professionals during discussions. In addition, the facilitators provided an opportunity for lived experience participants to share their views first before inviting others, which they were also free to decline.

##### Consensus focus group

For the consensus focus group, we invited professionals who previously took part in one of the three scoping focus groups, complemented with newly recruited professionals to ensure coverage across different disciplines.

### Data collection and analysis

#### Series of multistakeholder scoping focus groups

Prior to a focus group, all participants completed an online baseline questionnaire, including questions related to demographics (for both patients and professionals), pain experience (patients only), and professional experience (professionals only). We summarised participants’ demographics descriptively.

The HEIA-DH [19] structured the interview guide (Annexure C) and process, with further considerations drawn from the digital health equity framework [18], the Dahlgren-Whitehead model of health determinants [20], literature considering ‘digital’ as a new social determinant of health [67, 74] and other relevant literature ([35, 42, 63]). Where relevant, we adapted questions in the topic guide to the three priority groups, and refined the focus group structure and guide iteratively following the facilitators’ debrief session after the first two focus groups.

Focus groups were held virtually for two hours using Zoom. AC conducted focus groups discussions and AG, HC, SMA and SvdV facilitated group discussion. Except for SMA (identified as a man), all researchers identified themselves as women. Moreover, all researchers were post-doctorate digital health researchers, except for SMA and HC. All researchers were employed full time in their respective institutes. One of the facilitators (AG) captured key ideas emerging during the discussions on a digital whiteboard, visible to participants. Facilitators had background in health informatics, digital health equity, public health and conducting qualitative research. Facilitators discussed and developed key terms related to health equity in plain language and ensured the use of these defined terms throughout the group discussions. Also, participants from ethnic minorities could express their views in their local language, with one of the facilitators acting as a translator.

All focus groups were audio-recorded, transcribed verbatim by a professional transcription service, and anonymised by a researcher prior to analysis. We also included the facilitator’s notes on the whiteboard and anything participants shared via the chat function in the dataset for analysis.

We used a framework approach [29] as the HEIA-DH process provided a framework for coding. Initial codes were developed based on determinants of health, guided by the DigitALL model presented as part of the HEIA-DH [19], and key ideas emerging during discussions. HC and SMA iteratively refined the codes by reviewing all transcripts, digital whiteboards, and chats and applied the final version of the codebook to all textual data for consistency. These codes were grouped under four categories of the HEIA-DH to form an analytical framework, where columns were priority groups and rows were codes, elaborated through illustrative quotes. The four categories used in the analytical framework were: (a) social and digital determinants of health, (b) potential positive impacts, (c) potential negative impacts, and (d) mitigation strategies to manage impacts. We used the categories of the HEIA-DH; therefore, new conceptual ideas or codes were not developed. The HEIA-DH categories were used to compare participants’ views across three priority groups, for which the framework analysis approach was appropriate, as it allowed comparison without losing the context of individual participants’ views.

For impact-related categories (i.e., b and c), we presented priority sub-groups (i.e., sub-groups within priority groups) as identified by participants, either explicitly (i.e., mentioned by participants) or implicitly (i.e., implied from the priority group the focus group was about).

Research team members (AC, AG, HC, SMA, SNvdV) read and discussed the data, and discrepancies were resolved through team discussions. We used Microsoft Excel to manage all qualitative data, including the codebook and participants’ quotes.

##### Consensus focus group

Informed by the HEIA reporting checklist [57] and established value-based, multi-criteria prioritisation methods [31, 30, 70] participants in the consensus focus group selected mitigation strategies based on pre-defined criteria of ‘potential positive impact’ and ‘size of population affected’ as indicators of ‘overall value’ (i.e., improving digital pain equity of priority groups living with musculoskeletal pain conditions). Participants individually reviewed all strategies and rated them according to impact and population size (see annexure D). Participants’ ratings were summarised for all strategies during the focus group, which informed the subsequent group discussion where they selected which strategies they considered most likely to have value.

## Ethics approval and consent to participate

The study received an ethical approval from the University of Manchester’s Research Ethics Committee (ref # 2022-14094-23756).

## Results

### Study participants

A total of 28 people took part in the three scoping focus groups; Table 3 shows the characteristics of the 28 people who took part in the three scoping focus groups; demographics per focus group are presented in a supplementary table. Fifteen people were living with musculoskeletal pain conditions, and thirteen were professionals with expertise in areas such as chronic pain management, digital self-reporting tools, and health equity. Seven professionals attended the prioritisation focus group, six of whom took part in at least one of the prior scoping focus groups.

**Table 3:** Characteristics of participants

Characteristics of focus groups participants (*n* = 28)		
**Characteristics**	**Categories**	**Number (percentage)**
**People living with musculoskeletal pain conditions (*n* = 15)**		
Age	44 and younger	4 (27)
	45—64	5 (33)
	65 and older	6 (40)
Gender	Male	8 (53)
	Female	7 (47)
Ethnicity	White	9 (60)
	Asian or Asian British	4 (27)
	Black African/Caribbean	2 (13)
Socio-economic status	IMD; decile 1–3; most deprived	7 (46)
	IMD; decile 4–6;	4 (27)
	IMD; decile 7–10; least deprived	4 (27)
Employment status	Employed (full or part)	7 (47)
	Not working (unemployed; retired)	8 (53)
Number of musculoskeletal conditions	One	13 (87)
	Two or more	2 (13)
Number of other long-term conditions	1–2	9 (60)
	3 or more	6 (40)
Experience living with pain	1 to 3 years	6 (40)
	4 to 10 years	5 (33)
	More than 10 years	4 (27)
**Professionals with domain expertise (*n* = 13)**		
Age	25 – 44	8 (62)
	45—64	5 (38)
Gender	Male	6 (46)
	Female	7 (54)
Ethnicity	White	10 (77)
	Asian or Asian British	2 (15)
	Mixed/multiple ethnic group	1 (8)
Main profession/domain of expertise	Chronic pain researcher/expert	3 (23)
	Healthcare professional	3 (23)
	Technology researcher/developer	4 (31)
	Other	3 (23)
Years of professional experience	Less than a year	3 (23)
	4—10 years	6 (46)
	More than 10 years	4 (31)

*Abbreviations: IMD* Index of Multiple Deprivation, which is a relative measure of deprivation across small areas in the UK. The IMD is calculated based on socio-economic factors and living conditions. IMD deciles are constructed by ranking all small areas from most deprived to least deprived and dividing them into 10 equal groups [51]

### Multistakeholder scoping focus groups

After conducting a framework analysis of data from the three scoping focus groups, we developed a set of codes for each of the four categories informed by the HEIA-DH approach, which we describe in more detail below.

- Determinants of health associated with (digital) pain self-reporting

Table 4 below presents examples illustrating how social determinants may manifest in the context of digital health tools.

**Table 4:** Determinants of health relevant for pain equity

**Determinant of health**	**Examples of social determinants of health** *How social determinants may impact people’s perception, behaviour and attitude towards pain and pain management services*	**Examples of digital determinants of health** *How digital determinants may impact people’s use and adoption of digital pain self-reporting tools*
Age	*“there is an expectation of pain as you get older, and you accept that you will have pain”* (Male patient participant; FG with older adults)	*“It’s…to do with trust as well…some older adults will have a lot of fear built around using digital devices and giving away too much personal information”* (Researcher working with older adults; FG with older adults)
Gender	*“I think a lot of people might hesitate because you do get pain in those [private] areas but you don’t know how to explain, how to express”* (Female patient participant; FG with ethnic minorities)	
Ethnicity	*“[in certain cultures] because they would be basically disclosing how they feel to their family members….Once they disclose, then word gets out within the family very quickly and it puts them in a psychologically very weak state potentially”* (Male patient participant; FG with older adults)	
Social and community networks		*“so if you download the app but you get stuck is there somebody around you can go to get support with that”* (Researcher working with older adults; FG with older adults)
Language	*“I think a person's first language or language that they're most comfortable in can make an impact on [sort of] navigating health services”* (Female patient participant; FG with people living in deprived areas)	*“…a translation element of the digital solutions that could help those whose language isn't their first language English”* (Female patient participant; FG with people living in deprived areas)
Socio-economic status		*“..there a risk of increasing frustration by something like this tool [digital pain self-reporting] highlighting to you the inequalities in your own circumstances…….if you run out of data or your phone isn't very good”* (Female patient participant; FG with people living in deprived areas) *“So people with chaotic backgrounds, chaos within their lives, I think are very much marginalised….the last thing that they can afford usually is an internet”* (Female patient participant; FG with people living in deprived areas)
Privacy^a^		*“….you're in shared accommodation and you don't have privacy to sit and do it [digital pain self-report]”* (Female patient participant; FG with people living in deprived areas)
Confidentiality	*“the reason why people tick the 'Do not want to say' box is because they feel Big Brother's watching them and something negative is going to come out of being part of a research or ticking a box or giving information about themselves”* (Male patient participant; FG with ethnic minority group)	
Healthcare services	*“But that will stop people reporting [pain] because they have low expectations of results from their just verbal communications with medical professionals”* (Male patient participant; FG with older adults)	*“For some of the older adults they felt that being online was a negative loss of social interaction. So they enjoyed going to the doctors, they enjoyed the social connections that were made through face to face visits”* (Researcher working with older adults; FG with older adults)

*Abbreviations: FG* Focus Group

^a^Privacy is recognised as a digital determinant of health as privacy of digital data may impact the way health problems are understood, and health interventions are developed. [47, 48]

Participants also highlighted the intersectionality of their health determinants (i.e., multiple determinants simultaneously influencing equity impact of a digital pain self-reporting tool). For example, older adults lack trust in technologies, and they also perceive ‘being online’ as a loss of social interaction, effects of which are compounded if they have got additional needs due to their physical (e.g., dexterity) and mental health (e.g., depression).

(b) Potential positive impacts

Several potential positive impacts of digital pain self-reporting tools were highlighted in each scoping focus group. Table 5 outlines these impacts and illustrative quotes. In summary, participants thought digital pain self-reporting tools could enable people to capture their pain experience in real-time, help them communicate about their pain conveniently, and empower them to track and manage their pain effectively.

**Table 5:** Potential positive impacts of digital pain self-reporting tools to facilitate pain management

People who may benefit	Potential positive impact	Illustrative quote
People who lack self-efficacy	Increased pain awareness and self-efficacy	*“It gives you a little bit of autonomy and…ownership over your own pain…and ability to sort it”* (Female patient participant; FG with people living in deprived areas)
	Supporting pain self-management	*“it [could] potentially be a very powerful aid to self-care if you actually had access to the history of it. Because you have objective comparisons about how your pain was progressing and if as well at the same time you could put on some sort of contemporary note about your activities, be it dietary or physical, all of that empowers you as an individual to think about why has it got worse today, why was it better yesterday”* (Male patient participant; FG with older adults)
People with multiple co-morbidities	Supporting management of complex pain	*“….about the multi morbidity pain, so when people have pain in more than one part of the body I think the manikin can reflect that very well to a certain extent”* (Chronic pain researcher; FG with older adults)
Older people	Real-time pain self-reporting	*“it will make life easier for them [older people] because it will be the first place that we will go when we are having our pain”* (Female patient participant; FG with older adults)
People with language barriers	Aiding patient-provider communication	*“it has the potential to overcome some of the language barriers that you may see across the different ethnic groups, more for clinical practice I think rather than for research”* (Rheumatologist; FG with ethnic minorities)
People with limited financial resources	Remote monitoring opportunities	*“I was thinking about this idea of being able to show people remotely the symptom diary. Because it's potentially a way to reduce some of the gaps because if you don't actually have to come in to see the doctor to show them your symptom diary, that could actually save people travel money, getting a bus or a taxi”* (Female patient participant; FG with people living in deprived areas)
People with disability	Claiming public benefits	*“Potentially, can this app be used for helping people claim benefits and PIP [Personal Independence Payments]?”* (Female patient participant; FG with people living in deprived areas)
People experiencing issues with healthcare services	Recognition of a health problem	*“making it visible….that’s one of the main advantages about journeys to being diagnosed and getting their pain recognised by the medical profession”* (Female patient participant; FG with older adults)
	Addressing provider biases	*“I think it also has the potential to remove some of the conscious or unconscious bias that clinicians might have in hearing how people are describing their pain because it's a standard way of presenting the self-reported information”* (Rheumatologist; FG with ethnic minorities)
	Enriching communication	*“But actually people in pain, particularly chronic pain, tell us it’s not just my knee, it’s not just my back, it’s lots of other areas. So to be able to capture pain may be informative over and above just going to your GP with your app and saying this is what my pain is like…and it has a potential of enhancing health communication”* (Chronic pain researcher; FG with older adults)
	Active involvement in pain treatment	*“With my rheumatoid arthritis, I have to take a rituximab infusion every six to nine months. And by using this [digital pain self-reports] I can track quite easily when I'm ready for the next infusion because the intensity of pain increases and my mobility decreases…rather than just waiting for my consultant, ringing the rheumatology department up, asking for another blood test and then arranging an infusion to manage the pain better”* (Male patient participant; FG with ethnic minorities)

*Abbreviations: FG* Focus Group

(c) Potential negative impacts

Participants described a range of potential negative impacts of using digital pain self-reporting tools to facilitate pain management, which could create new or exacerbate existing inequities in pain. In general, they considered acquiring pain information from digitally excluded people a challenge. In addition, incorrect interpretation of pain self-reports might impact decision making negatively. For example, if non-reporting days are incorrectly interpreted as pain free days, this may trigger an unwarranted change in treatment plan, which may further marginalise these priority groups. For example, one participant said:

I was having a day that for me is okay but that might not necessarily be that I wasn't in pain because my pain is there all the time. And if then a clinician was to look at that and interpret that as, oh, she's doing better now, you know, they might try to reduce my meds (Female patient participant; FG with older adults)

We present a brief overview of these potential negative impacts along with illustrative quotes in Table 6.

**Table 6:** Potential negative impacts of digital pain self-reporting tools to facilitate pain management

People who would be at risk	Potential negative impact	Illustrative quote
People with different ethnic background	Exclusion from digital health research	*“if some ethnic groups have less access to the technology or the connectivity, then they may be less represented [in research]”* (Researcher; FG with ethnic minorities)
	Pain perceived as a weakness	*“Depending on cultural background, there is a huge difference in mindset, certain cultures don't like acknowledging pain because they actually deem it as a weakness…and if they do acknowledge it, if they are brought out of their comfort zone…does it make them feel weaker mentally and emotionally as well”* (Inclusion expert; FG with ethnic minorities)
	Gender stereotyping	*“And it's a gender issue as well of women going, oh, yeah, pain, that's just life, put up with it”* (Female patient participant; FG with ethnic minorities)
	Lack of knowledge of pain terminologies	*“Because I think culturally, some patients are lost because they don't know the difference between sharp pain and dull pain”* (Male patient participant; FG with ethnic minorities)
People of older age	Fear of losing face-to-face interaction with clinicians	*“…what you've not got is, you've not got that interaction, which is not necessarily a downside but I think sometimes you need that interaction with somebody there because a certain pain report might be quite alarming to a clinician, for whatever reason, and the patient may not know why but you would sort of instantly have some follow-up questions”* (Older male participant; FG with older adults)
People living in socio-economically deprived areas	Emphasis on one’s limitations	*“Using the app [digital pain self-reports] could make you feel just more isolated because it might highlight your inadequacies, you know, like dyslexia…because I do struggle with that…[which] sort of damage your self-esteem”* (Female patient participant; FG with deprived areas)
People with pain related anxiety	Too much emphasis on pain	*“This [pain self-reporting] is making me think about my pain more and I started to wonder if that's possibly a bad thing”* (Female patient participant; FG with deprived areas)

*Abbreviations: FG* Focus Group

(d) Strategies to mitigate the effects of potential negative equity impacts

Participants in the multi-stakeholder focus groups proposed a wide range of 32 mitigation strategies to prevent, reduce or eliminate the potential negative impacts of using digital pain self-reporting tools on health equity. We categorised these proposed strategies into four broader categories, which we summarise below. The full list of strategies is included in the annexure D.

i. Facilitate digital access and skills to enable pain self-reporting

To facilitate access to digital pain self-reporting tools, their compatibility to different operating systems (e.g., Android, iOS, Windows) and their availability on larger screen sizes (e.g., laptop for older adults) are important considerations. People with low socio-economic background could be supported through the provision of digital devices at public places (e.g., libraries, clinics of general practitioners) with a digital pain self-reporting tool installed on them or by collaborating with community-based organisations. These approaches could improve people’s physical access to digital devices and other resources to enable those with poor digital skills to get support (such as peer support, helpline support) in using these tools for pain self-reporting.

ii. Improve the ease-of-use and relevance of pain self-reporting tools

Participants had different suggestions for how to change digital pain self-reporting tools to manage negative equity impacts. For example, for people with language barriers, tools could offer translation support (including glossary of pain terminologies), and for people with physical, sensory and cognitive conditions additional accessibility features (e.g., higher contrast, font size, etc.) and design considerations (e.g., consistency in design) were mentioned.

Looking at the Manchester Digital Pain Manikin app as an example of a digital tool, participants suggested improvements of the manikin image (e.g., by adding lateral sides) and emphasised the need for it to be customisable (e.g., gender personalisation). Participants also proposed more engaging and less text-heavy ways of pain self-reporting (e.g., pictograms for reporting pain types).

iii. Supporting materials to aid completion and interpretation of pain self-reports

Participants suggested using digital pain self-reporting data to help people manage their condition better, for which providing guidance on interpreting pain self-reports were considered important for improving people’s pain self-awareness and self-management. Similarly, participants proposed capacity-building activities for increasing healthcare professionals’ knowledge of pain and to reflect on their attitudes towards pain to support improved clinical decisions based on the summaries of pain self-reports.

Participants also made suggestions for how to improve people’s digital skills in general and of digital pain self-reporting in particular. For example, by developing tutorials to improve basic digital literacy (e.g., how to use the internet), and providing easy-to-understand instructions in written and video format to explain how to use digital pain self-reporting tools.

iv. Building trust in pain reporting and health technology and research

Lastly, strategies were proposed for building trust in health technology and research. Making a digital pain self-reporting tool password protected; explaining to users how their data collected via these tools would be used; and giving them control over which data to share and with whom could help engender this trust.

Seven experts (including healthcare professionals, researchers, experts) participated in the consensus focus group and selected 20 strategies to take forward for managing the potential negative impact of digital pain self-reporting tools on priority groups’ health equity (Table 7). Based on participants’ own professional background and experience of working with priority groups, they argued overall value of strategies, rather than against any strategy. Participants also identified the need of returning to those strategies which are not selected because of ratings and group discussions.

**Table 7:** Selected strategies to mitigate potential negative impacts

Facilitate digital access and skills to enable pain self-reporting
Ability to use digital pain self-reporting tools across platforms (e.g., iOS, android) and devices (e.g., smartphone, tablet, computer)
Partner with community organisations to facilitate access to the Internet and devices for people who cannot afford a computer, smartphone or data to allow them to submit pain self-reports
Organise peer support to encourage and help people with using a digital device for pain self-reporting
Offer helpline support to help people with using digital pain self-reporting tools
Improve the ease-of-use and relevance of pain self-reporting tools
Offer zoom-in options for people with visual or dexterity impairments to read instructions or interact with digital pain self-reporting tools
Develop a glossary of culturally attuned pain terminologies for ethnic minority groups
Enable customisation of notifications and reminders for increasing completeness of pain self-reports
Add lateral views to manikin-based pain self-reporting tools to enhance accuracy of reporting of pain location
Enable customisation of a manikin's appearance to align with users’ personal characteristics and preference (e.g., skin tone, gender, body shape)
Enable reporting of location-specific pain types (such as shooting pain, burning), for example by using pictograms to support diagnosis and assessment of treatment response
Supporting materials to aid completion and interpretation of pain self-reports
Develop easier to understand written user instructions about how to complete pain self-reports
Offer pain questions and instructions in other languages rather than in English only
Develop user instructions about how to complete pain self-reports in non-written formats, e.g., short videos or audio
Develop guidance or training for patients on how to interpret pain self-reports (e.g., how to distinguish good from bad days)
Develop guidance or training for healthcare professionals on how to interpret pain self-reports
Building trust in pain reporting and health technology and research
Build trust in research by explaining how data will be used (e.g., in a within-tool data privacy statement)
Protect digital devices (especially mobile devices) and applications with a password or biometric authentication to prevent unauthorised access to people’s pain reports
Enable users to control what part of their pain reports they share and with whom
Engage with patients and community gatekeepers to communicate how pain self-reports can help them with pain management
Develop testimonials, posters and case studies to encourage people to use digital pain self-reporting tools

## Discussion

### Summary of findings

We utilised the HEIA-DH approach to identify equity considerations for digital pain reporting tools with a focus on three priority groups, including older adults, ethnic minorities and people living in deprived areas. We found that these priority groups could benefit from digital pain self-reporting tools through capturing pain information in real-time to facilitate pain management; addressing provider biases; and enriching patient-provider communication. Whilst these tools are developed to enhance pain management, they may also negatively impact on certain groups, albeit unintended. Exclusion from these tools may be due to, for example, language barriers, learning difficulties, and/or lack of comfort with technology; others may experience negative effects because of too much emphasis on pain. To manage these negative equity impacts, we identified 20 strategies across four categories: (a) facilitate pain self-reporting through within-tool changes (e.g., offer zoom-in options for people with visual or dexterity impairments); (b) facilitate pain self-reporting by improving digital skills and physical access to digital resource (e.g., develop easier-to-understand written user instructions about how to complete pain self-reports); (c) developing approaches for increasing the value of pain self-reports (e.g., developing materials to help healthcare professionals to better interpret pain self-reports); and (d) building trust in technology and research (e.g., enable users to control what part of their pain reports they share and with whom).

### Relation to other studies

In the recent past, equity frameworks have been developed to describe health equity impacts of digital health ([18], Richardson et al., 2022; [41, 45]). These frameworks have been applied to virtual care and mobile personal health records [76], but not to digital self-reporting tools for pain assessment and management. In this study, we took a qualitative assessment approach by involving multiple stakeholders, whereas for assessing health equity impact of mobile personal health records a case study approach was adopted, without involving (or mentioning) end users and relevant stakeholders [76].

In our study, the emphasis on usability and user requirements of digital pain self-reporting tools was driven by diverse characteristics of people living with chronic pain. Ensuring ease-of-use and fulfilling user requirements were considered important determinants of user engagement with digital pain management tools or interventions ([12, 13, 33]). For example, better engagement with a digital care program translated into better pain management outcomes across all five groups developed based on social deprivation index [10]. Additionally, improving user engagement (accounting for diverse characteristics) may help in achieving scalability of digital health intervention and health equity in pain management [32].

In our study, we presented equity considerations for sub-groups (e.g., older age and socio-economic deprivation being considered for ethnic minorities) within priority groups in terms of potential positive and negative impacts and strategies to manage them. This suggests intersectional nature of the inequities, i.e., individuals grouped as a priority group may have different needs, some of which may align with needs of other priority groups. Such considerations for individuals are currently missing in the existing literature related to health inequities [34]. Moreover, in addition to recognising such differences at individual and community levels, achieving digital health equity also means operationalising equity considerations at a system level [41, 45]. Such considerations may translate into having a diverse clinical team, which might be one of the potential reasons in achieving better pain management outcomes in a digital intervention [10]. In addition to such differences, there is a wider recognition of engaging with communities and improving their digital skills in addressing digital health inequities ( [24, 37, 62]).

Participants in our study suggested that digital pain self-reporting may have a positive impact on people’s pain awareness and self-efficacy, which aligns with findings reported by a study exploring people’s experiences of using an app-based cognitive-behavioural pain self-management intervention [13]. Similarly, we found in our study that the daily pain-self-reporting may make people feel anxious, which is also reported by Bhattarai et al. [12] for digital pain self-management among older adults. However, our approach also allowed us to capture potential strategies to manage these impacts, such as engaging with patients and community gatekeepers to communicate how pain self-reports can help them with pain management.

### Limitation

Our study has some limitations. Firstly, focus groups were conducted virtually which might have excluded those who are less likely to use digital technologies, thus overlooking perspectives of those who experience the greatest impacts of digital determinants of health. However, previous researcher suggested that virtual focus groups can be effective [8, 52] by addressing participation barriers related to factors such as travel, childcare needs, and difficulty to take time off work, etc.

Secondly, only one focus group was conducted with each priority group, which although a source of important input, may not have allowed for a full range of perspectives or views across the diverse groups. Additional focus groups with each priority groups could provide more robust and insightful analyses, including aspects related to the intersection of identities within and across groups and how this may impact health equity.

Lastly, the HEIA-DH approach does not provide a method for prioritising among proposed mitigation strategies. We used a purposively chosen group of experts to support selection of strategies; this was facilitated by having members rate the potential size of the impact and size of the impacted population. This is ultimately a utilitarian approach that may inadvertently erase true consideration of equity, which by definition may be applicable to smaller subgroups rather than impacting the majority. Further, our consensus group for prioritisation was made up of only domain experts and was planned post-hoc due to the unanticipated large number of responses gathered during the three focus groups. Thus, participation from people with lived experience was not planned or consented to attend consensus focus group.

### Implications

This study has highlighted implications in two areas: (a) future development of digital pain self-reporting tools and considerations to building support and resources around their use, and (b) improving methods to assess equity impacts of digital pain self-reporting tools and identify strategies to manage these impacts.

Despite digital pain self-reporting and management tools being widely available [5, 4, 77] and strong arguments supporting their efficacy [69, 71], health equity is rarely considered as part of their development and evaluation [32]. Developers of digital pain self-reporting tools should consider implementing the strategies or equity considerations identified in our study, where relevant to their priority group(s) and even beyond these groups. For example, manikin’s personalisation and views (i.e., front, back, left, right) identified in our study were also considered important for the cross-cultural acceptability of digital pain self-reporting tools [3, 6]. Therefore, future research into monitoring the implementation of these equity and intersectional considerations and their impact on user engagement and pain management outcomes should be considered.

In addition to equity considerations, digital health researchers and developers should adopt new methods or approaches to prioritise user requirements gathered from a variety of priority groups. In our study, we used an equity-driven approach to prioritise user requirements, because implementing user requirements to address digital pain inequities at once is pragmatically challenging [15]. Further challenge is posed by finding a balance between user requirements and feasibility of transforming them into design of a digital tool. However, user requirements to ensure potential positive impacts of digital pain self-reporting tools should be considered first because they are easier to achieve [26], while negative impacts can be addressed simultaneously. This can be supported by incorporating a HEIA reporting checklist [57], which can be operationalised to monitor and evaluate the health equity impacts [57, 66] of digital pain self-reporting tools and optimising pain management. Moreover, accounting for the existing health inequities in appraising health technology assessment methods is also acknowledged by the National Institute for Health and Care Excellence [9]. Therefore, further application of equity-driven approach and discussions are necessary to explore the equity of approaches to prioritisation and selection as well as to evaluate health equity impact of digital pain self-reporting tools.

## Conclusion

Our study identified considerations relevant for developing equitable digital pain self-reporting tools and resources and skills to support adoption and use of these tools among priority groups. We also identified sub-groups within priority groups who may have their own, specific equity needs regarding digitally enabled pain assessment and management. This warrants methods and approaches that consider equity and intersectionality when gathering, selecting, or implementing user requirements for digital pain self-reporting tools. Future research should focus on implementing the strategies identified by our study, as and when relevant to the priority group of interest and monitor the impact of this on health equity of people living with chronic pain. Ultimately, this will help optimising pain management outcomes across the population, including priority groups.

## Supplementary Information

Supplementary Material 1.

Supplementary Material 2.

Supplementary Material 3.

Supplementary Material 4.

Supplementary Material 5.

## Data Availability

Data is provided within the manuscript or supplementary information files.
