# Supplementary material for: Strategies to optimise the health equity impact of digital pain self-reporting tools: a series of multi-stakeholder focus groups
Source: Int J Equity Health. 2024 Nov 11;23:233. doi: 10.1186/s12939-024-02299-w (PMC11555918; doi:10.1186/s12939-024-02299-w)
Supplement: Supplementary file 2 — Supplementary Material 2. [file 12939_2024_2299_MOESM2_ESM.pdf]

ASSESSING AND ADDRESSING HEALTH EQUITY BARRIERS FOR DIGITAL PAIN SELF-REPORTING

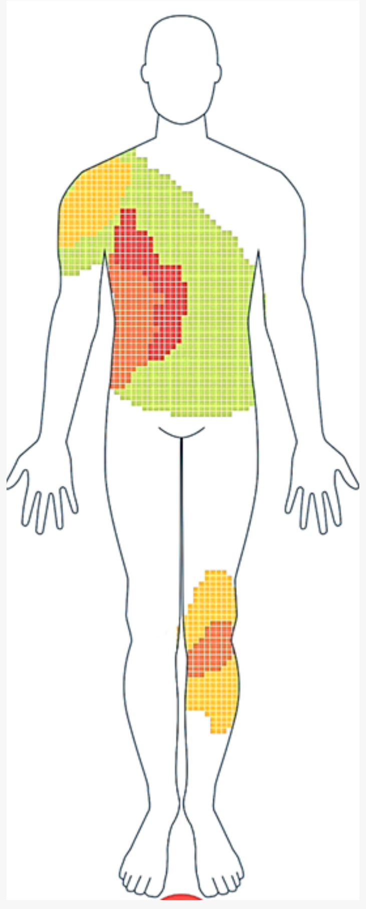

DO YOU HAVE ARTHRITIS OR ANY OTHER LONG-TERM MUSCULOSKELETAL PAIN CONDITION?

ARE YOU INTERESTED IN IMPROVING THE WAY HOW LONG-TERM PAIN IS MEASURED?

We are looking for people with experience of living with a painful musculoskeletal condition to attend a 2-hour focus group discussion. Discussions will be able to understand the impact of introducing digital self-reporting on health equity

ARE YOU?
- AN ADULT (AGED 18 OR ABOVE)
- AFFECTED BY ARTHRITIS OR ANY OTHER LONG-TERM MUSCULOSKELETAL PAIN CONDITION
- ABLE TO SPEAK, READ AND UNDERSTAND ENGLISH
- CONNECTED TO THE INTERNET?

THEN

HELP US!

BY EXPRESSING YOUR INTEREST FOR AN ONLINE FOCUS GROUP DISCUSSION

TO EXPRESS YOUR INTEREST IN TAKING PART PLEASE
CONTACT US AT:
PAINMANIKIN@MANCHESTER.AC.UK

MANCHESTER 1824
The University of Manchester

The Manchester Digital Pain Manikin Study Flyer
Version 1.0., 20/04/2022

Watch orientation video: https://www.youtube.com/watch?v=Ol32C3VzM7A

You will be compensated for your time
