# Supplementary material for: Strategies to optimise the health equity impact of digital pain self-reporting tools: a series of multi-stakeholder focus groups
Source: Int J Equity Health. 2024 Nov 11;23:233. doi: 10.1186/s12939-024-02299-w (PMC11555918; doi:10.1186/s12939-024-02299-w)
Supplement: Supplementary file 3 — Supplementary Material 3. [file 12939_2024_2299_MOESM3_ESM.docx]

# **Annexure C**

[Specific priority group*]

Semi-Structured Focus Group Guide

Focus group structure:

Note: There is a 10 min break, but participants can take additional breaks if they feel the need.

| Topic and timings  (2 hours total) | Questions and prompts |
| --- | --- |
| Introductions and overview of agenda  15 min   - Introductions from the team - Allow each participant to introduce themselves - Review purpose of meeting and agenda; ground rules - Brief presentation that review the Manchester Digital Pain Manikin (MDPM) - Definitions of health equity; digital health equity; other relevant terms - Overview of the HEIA-DH process | |
| Scoping  *The scoping phase is used to identify any groups or populations that the Manchester Digital Pain Manikin (MDPM) may impact.*  20 min | Provide background for why the *[disadvantaged group]* was chosen as a focus for improving digital health equity?  Manchester Digital Pain Manikin (MDPM).  Present any literature findings relevant to this group in general with respect to:  Which social determinants are relevant for this group?  Questions for focus group:  “Are there any health equity or socioeconomic factors that we have overlooked for *[disadvantaged group]*?”  “Are there any intersectional factors that we have overlooked that are important for *[disadvantaged group]* ?  “Which are the most important health equity factors impacting *[disadvantaged group]*?”  “How do these socioeconomic factors shape digital determinants of health for *[disadvantaged group]*?”  [Prompts: any impacts in the areas of: infrastructure and resources, technology, privacy and security, culture and beliefs about either technology or pain, digital or health literacy, language]  “Is there anyone else who should be involved in this conversation or planning?” |
| Potential impacts – both positive and negative  *Assess the unintended impacts of the MDPM for this population. This should include consideration of strengths as*  *well as challenges that might be experienced by individuals and families within each group.*  25 min | Given the social and digital determinants of health for *[disadvantaged group]* identified in step 1:  **Present findings from feasibility study relevant to** *[disadvantaged group]*  “What are the possible **positive impacts** of the Manchester Digital Pain Manikin on these equity factors for *[disadvantaged group]*?”  “What strengths, skills, and resources that *[disadvantaged group]* bring to utilising the MDPM?”  “What are the **unintended negative impacts** of the Manchester Digital Pain Manikin on these equity factors for *[disadvantaged group]*?”  “Will this impact access to care and / or the ability to achieve equal outcomes from care for *[disadvantaged group]*?”  **Prompts: Strengths and needs in areas of:**  infrastructure and resources  technology privacy and security  culture and beliefs about either technology or pain digital or health literacy  language  providers/ health care professionals  organisational level  systems level (funding, policy etc.)  “Of these factors which are the most important to address for *[disadvantaged group]*?”  “Are there any areas where more information is needed?” |
| BREAK 10 MIN | |
| Mitigation strategies  *Using the potential impacts identified, develop an evidence-based mitigation*  *strategy, including evidence specific for this population.*  25 min | The research team will summarise and rank the impacts for *[disadvantaged group]* described by the group. We will focus on 3 negative impacts and 1-2 positive impacts  For each impact:   “What are steps or actions that could be taken to address this impact?” |
| Monitoring  15 min | The research team will summarise and rank the mitigation strategies described by the group, and identify 3-4 for focus.  For each of the mitigation strategies:  “How will we know that the action or step we have taken is successful at improving the equity of the MDPM for this population?”  “Are there tools we can use to measure this?” |
| Closing | Thank participants |
