# Supplementary material for: Strategies to optimise the health equity impact of digital pain self-reporting tools: a series of multi-stakeholder focus groups
Source: Int J Equity Health. 2024 Nov 11;23:233. doi: 10.1186/s12939-024-02299-w (PMC11555918; doi:10.1186/s12939-024-02299-w)
Supplement: Supplementary file 4 — Supplementary Material 4. [file 12939_2024_2299_MOESM4_ESM.docx]

# **Annexure D**

# Methodological details of fourth focus group and a complete list of strategies

We conducted a final, fourth online focus group to prioritise mitigation strategies identified in the first three sessions.

Informed by established value-based, multi-criteria prioritisation methods, our prioritisation approach considered the ‘potential positive impact’ and ‘size of population affected’ for each strategy. Together, these two criteria allowed rating each mitigation strategy based on its likelihood to create an overall value, i.e., improving digital pain equity of priority groups living with musculoskeletal pain conditions in the UK. The higher the overall value of a strategy, the stronger the recommendation for incorporating the strategy into digital pain self-reporting tools.

We started the session with briefly summarising the findings of focus group discussions, defining key terminologies, and explaining the prioritisation process to all participants. Participants then rated all mitigation strategies as low, medium or high for the criterion of ‘potential positive impact’ and small, medium or large for the criterion of ‘size of population affected’. All ratings were summarised in a matrix (Figure 1). We assigned a high priority to strategies where more than 50% of participant ratings were positioned in the ‘high priority’ cells in the matrix, i.e., strategies which participants considered to potentially create medium-to-high positive impact for a medium-to-large size of population. Similarly, we assigned low priority if more than 50% of participant ratings ended up in the ‘low priority’ cells. Strategies where the majority of ratings were in the ‘?’ cells or where ratings were spread across matrix areas (i.e., where there was no clear majority) were discussed by the group to see if a consensus on the strategy’s priority could be reached; if no consensus was reached, we considered the strategy of ‘unknown priority’.

**Figure 1:** Strategy-specific matrix for summarising ratings

| **Name of a proposed mitigation strategy** | | | | |
| --- | --- | --- | --- | --- |
| **Size of population affected** | *Small* | Low | Low | ? |
|  | *Medium* | Low | ? | High |
|  | *Large* | ? | High | High |
|  | | *Low* | *Medium* | *High* |
|  |  | **Potential positive impact** | | |

**Key decisions:**

***Consider for the technology improvement:*** Medium-to-high impact affecting medium-to-large populations (grids shown in green with ‘High’)

***Dismiss for the technology improvement until the next evaluation:*** Low-to-medium impact affecting small-to-medium populations (grids shown in red with ‘Low’)

***Discuss until consensus is achieved:*** High impact affecting small population, medium impact affecting medium population, and low impact affecting large population (grids shown in yellow with ‘?’)

Using a strategy-specific matrix for all strategies, a complete list of strategies was prepared as given below:

| Facilitate digital access and skills to enable pain self-reporting | Priority |
| --- | --- |
| Ability to use digital pain self-reporting tools across platforms (e.g., iOS, android) and devices (e.g., smartphone, tablet, computer) | Yes |
| Partner with community organisations to facilitate access to the Internet and devices for people who cannot afford a computer, smartphone or data to allow them to submit pain self-reports | Yes |
| Organise peer support to encourage and help people with using a digital device for pain self-reporting | Yes |
| Offer helpline support to help people with using digital pain self-reporting tools | Yes |
| Enable offline use of digital pain self-reporting tools for people without regular access to the Internet | No |
| Apply consistent design especially across mobile app screens to reduce cognitive burden of learning to use digital pain self-reporting tools | No |
| Access manikin-based pain self-reporting tools on digital devices with a larger screen for people with visual impairment | No |
| Offer (customisation of) colour contrasts for people with visual impairment and learning difficulties | No |
| Develop/signpost to guidance for people to learn about using the internet and digital devices (e.g., computer, smartphone, tablets) for pain self-reporting | No |
| Improve the ease-of-use and relevance of pain self-reporting tools |  |
| Offer zoom-in options for people with visual or dexterity impairments to read instructions or interact with digital pain self-reporting tools | Yes |
| Develop a glossary of culturally attuned pain terminologies for ethnic minority groups | Yes |
| Enable customisation of notifications and reminders for increasing completeness of pain self-reports | Yes |
| Add lateral views to manikin-based pain self-reporting tools to enhance accuracy of reporting of pain location | Yes |
| Enable customisation of a manikin's appearance to align with users’ personal characteristics and preference (e.g., skin tone, gender, body shape) | Yes |
| Enable reporting of location-specific pain types (such as shooting pain, burning), for example by using pictograms to support diagnosis and assessment of treatment response | Yes |
| Offer the option of submitting a briefer pain report (reflecting a minimum pain data set) to reduce reporting burden on days of intense/impactful pain | No |
| Copy-paste previous pain reports to save time e.g., if pain has not changed (substantially) | No |
| Offer voice recognition to input responses (e.g., for people with visual impairments or low literacy) | No |
| Offer audio to listen to questions (e.g., for people with visual impairments or low literacy) | No |
| Enable uploading of pictures taken with smartphone as part of pain report to illustrate and share pain experience (e.g., photo of swollen joints in the hand) | No |
| Develop a 3-dimensional manikin for manikin-based digital pain self-reporting tools to enhance accuracy of reporting of pain location | No |
| Supporting materials to aid completion and interpretation of pain self-reports |  |
| Develop easier to understand written user instructions about how to complete pain self-reports | Yes |
| Offer pain questions and instructions in other languages rather than in English only | Yes |
| Develop user instructions about how to complete pain self-reports in non-written formats, e.g., short videos or audio | Yes |
| Develop guidance or training for patients on how to interpret pain self-reports (e.g., how to distinguish good from bad days) | Yes |
| Develop guidance or training for healthcare professionals on how to interpret pain self-reports | Yes |
| Provide guidance on how people can use pain reports for accessing health and social services, e.g., as evidence for a request for public benefits | No |
| Building trust in pain reporting and health technology and research |  |
| Build trust in research by explaining how data will be used (e.g., in a within-tool data privacy statement) | Yes |
| Protect digital devices (especially mobile devices) and applications with a password or biometric authentication to prevent unauthorised access to people’s pain reports | Yes |
| Enable users to control what part of their pain reports they share and with whom | Yes |
| Engage with patients and community gatekeepers to communicate how pain self-reports can help them with pain management | Yes |
| Develop testimonials, posters and case studies to encourage people to use digital pain self-reporting tools | Yes |
