# Supplementary material for: Strategies to optimise the health equity impact of digital pain self-reporting tools: a series of multi-stakeholder focus groups
Source: Int J Equity Health. 2024 Nov 11;23:233. doi: 10.1186/s12939-024-02299-w (PMC11555918; doi:10.1186/s12939-024-02299-w)
Supplement: Supplementary file 5 — Supplementary Material 5. [file 12939_2024_2299_MOESM5_ESM.docx]

**Supplementary table:** Characteristics of participants for each priority group

| Characteristics | Categories | Older adults (n=5) | Ethnic minorities (n=6) | People living in deprived areas (n=4) |
| --- | --- | --- | --- | --- |
| Age | 44 and younger | 0 | 4 | 0 |
|  | 45 - 64 | 0 | 2 | 3 |
|  | 65 and older | 5 | 0 | 1 |
| Gender | Male | 4 | 3 | 1 |
|  | Female | 1 | 3 | 3 |
| Ethnicity | White | 5 | 0 | 3 |
|  | Asian or Asian British | 0 | 3 | 1 |
|  | Black African/Caribbean | 0 | 3 | 0 |
| Socio-economic status | Index of Multiple Deprivation; decile 1-3 | 1 | 3 | 3 |
|  | IMD; decile 4-6 | 1 | 2 | 1 |
|  | IMD; decile 7-10 | 3 | 1 | 0 |
| Employment status | Employed (full or part) | 1 | 3 | 3 |
|  | Not working (unemployed; retired) | 4 | 3 | 1 |
